# Racial/Ethnic Differences in the Longitudinal Effects of Fear of Falling on Falls

**DOI:** 10.1093/geroni/igab046.3163

**Published:** 2021-12-17

**Authors:** Tuo Yu Chen, Giyeon Kim

**Affiliations:** 1 Taipei Medical University, Taipei, Taipei, Taiwan (Republic of China); 2 Chung-Ang University, Seoul, Seoul-t'ukpyolsi, Republic of Korea

## Abstract

Research suggests that the effects of fear of falling on falls may differ by race/ethnicity. We investigated whether race/ethnicity (White, Black, and Hispanic) moderated the longitudinal effects of fear of falling on the incidence of falling and having a repeated fall among community-dwelling older adults. We used data from 2011-2018 of the National Health and Aging Trends Study (NHATS). These included a total of 19,516 person-intervals from 5,113 respondents. Self-reported any fall in the past year was the outcome variable with baseline fear of falling as the predictor and race/ethnicity as the moderator. Known risk factors for falls were included as covariates. Results showed that among respondents without the experience of falling at baseline, baseline fear of falling significantly increased the odds of a new-onset of fall at 1-year follow-up among Blacks, compared to Whites. Among respondents who already fell at baseline, baseline fear of falling significantly increased the odds of having a repeated fall later on among Hispanics, compared to Whites. Clear evidence of racial/ethnic differences was found in the relationship between fear of falling and falls among community-dwelling older adults in the U.S. Special attention should be paid to Black older adults with a fear of falling but have not fallen down recently and Hispanics with fear of falling and have fallen in the past year. Readily available educational programs should be actively advertised to older adults to reduce the fear of falling and culturally tailored educational programs should be developed for older adults from racial/ethnic minority backgrounds.

